# A narrative review of research advancements in pharmacogenetics of cardiovascular disease and impact on clinical implications

**DOI:** 10.1038/s41525-025-00511-6

**Published:** 2025-07-10

**Authors:** Ayat Shorbaji, Peter Natesan Pushparaj, Ayat B. Al-Ghafari, Loubna Siraj Mira, Mohammad Abdullah Basabrain, Muhammad Imran Naseer, Farid Ahmed, Muhammad Abu-Elmagd, Mahmood Rasool, Sherin Bakhashab

**Affiliations:** 1 Department of Biochemistry, Faculty of Sciences, King Abdulaziz University, Jeddah, Saudi Arabia; 2 Institute of Genomic Medicine Sciences, King Abdulaziz University, Jeddah, Saudi Arabia; 3 Experimental Biochemistry Unit, King Fahd Medical Research Centre, King Abdulaziz University, Jeddah, Saudi Arabia; 4 Department of Medical Laboratory Technology, Faculty of Applied Medical Sciences, King Abdulaziz University, Jeddah, Saudi Arabia

**Keywords:** Cardiovascular diseases, Drug discovery

## Abstract

Pharmacogenetics can enhance cardiovascular disease (CVD) treatment by tailoring drug therapy to genetic profiles and minimising trial-and-error approaches. Genetic variability influences responses to common CVD drugs, including antiplatelet drugs (clopidogrel and aspirin), anticoagulants (warfarin), statins, and antihypertensives (ACE inhibitors and β-blockers). Understanding genetic polymorphisms can improve efficacy and safety. Despite this progress, further research is needed to optimise pharmacogenomic applications and advance personalised medicine to improve CVD treatment outcomes.

## Introduction

Cardiovascular disease (CVD) is the leading cause of mortality globally, with the number of deaths increasing from 12.1 million in 1990 to 20.5 million in 2021^1^. Pharmacogenetics is a relatively new area of pharmacology that focuses on pharmacogenetic factors that can significantly improve clinical outcomes by reducing side effects and increasing the efficacy of medications used to treat CVD. Therefore, understanding the impact of genetic polymorphisms on the pharmacokinetics and pharmacodynamics of therapeutic agents is imperative for healthcare practitioners^2^.

Efforts to improve the therapeutic efficacy of currently available drugs have been made through pharmacogenetic research, and the findings suggest the value of developing new drugs based on the association of variations in genes critical for drug action with variable outcomes of drug therapy. These variants often have smaller individual effect sizes and can determine the variability in drug responses in an individual or across a population. Thus, the primary objective of this review is to provide a narrative synthesis of the current knowledge on the pharmacogenetics of CVD drugs, with a dual focus on summarising key research findings and offering guidance on the clinical applicability of genetic markers. By exploring genetic polymorphisms that influence drug metabolism, transport, and therapeutic targets, this review aims to: (1) Highlight the most clinically actionable genetic polymorphisms of certain genes such as *CYP2C19*, *VKORC1* and *SLCO1B1*, which have demonstrated significant impacts on drug efficacy and safety in the context of CVD therapy; (2) Discuss emerging genetic variants that remain within the research domain and evaluate their potential to transition into clinical practice; (3) Examine real-world challenges, including controversies, limitations, and barriers to implementing pharmacogenetic testing in routine CVD management; and (4) Provide actionable insights into how pharmacogenetic testing can be integrated into clinical workflows to improve personalised medical outcomes for patients with CVD.

## The pharmacogenetics of antiplatelet therapy

### The pharmacogenetics of purine receptor antagonists

Patients with acute coronary syndrome (ACS) or stroke and those undergoing percutaneous coronary intervention (PCI) are treated with oral antiplatelet therapy, such as ticagrelor, prasugrel and clopidogrel^3,4^.

#### The therapeutic target of thienopyridine and non-thienopyridine family drugs

The therapeutic target of these drugs is to act against platelets by blocking the purine receptor P2Y12 (P2RY12), which prevents adenosine diphosphate (ADP) from stimulating platelet aggregation^5^. Clopidogrel is the most frequently used antiplatelet P2Y12 inhibitor. Clopidogrel combined with aspirin is a well-recognised dual antiplatelet therapy to prevent ischaemic cardiovascular events^3^.

#### The metabolism of thienopyridine and non-thienopyridine family drugs

**Fig. 1:**
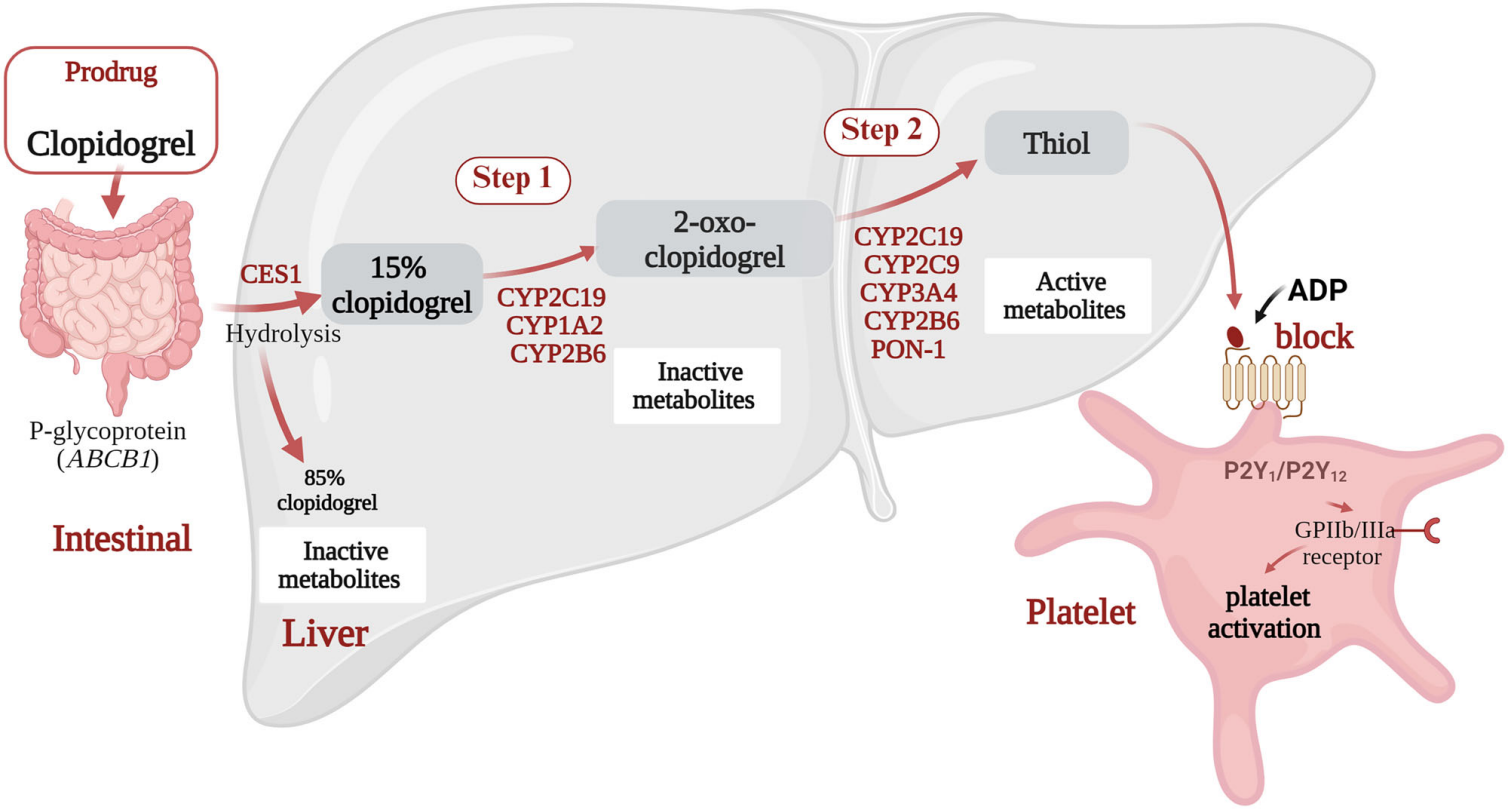
Mechanism of action of clopidogrel. The intestinal absorption of clopidogrel is restricted by P-glycoprotein (P-gp), which is encoded by *ABCB1*. Carboxylesterase 1 (CES1) hydrolyses 85% of its uptake into inactive metabolites. Only 15% of inactive clopidogrel is oxidised by liver-resident cytochrome P450 (CYP450) enzymes to 2-oxo-clopidogrel, which is also inactive. Subsequently, CYP450 and paraoxonase enzymes convert 2-oxo-clopidogrel into active metabolites (thiols), which bind to the P2Y12 receptor on the platelet surface. This binding prevents the activation of the GPIIb/IIIa complex by ADP, which inhibits platelet activation (modified from refs. ^6,7^). Figure created with Bio Render.com.

Clopidogrel is a second-generation prodrug of the thienopyridine family^5^. The transport of clopidogrel to the intestinal tract and its conversion into active components by various enzymes in the liver is necessary for its therapeutic effect. The absorption of clopidogrel in the small intestine is regulated by P-glycoprotein (P-gp) encoded by the ATP-binding cassette subfamily B member 1 (*ABCB1*) gene. Approximately 85% of the absorbed clopidogrel in the intestine is hydrolysed to inactive metabolites by carboxylesterase 1 (CES1) and then eliminated in the urine or faeces. Approximately 15% of clopidogrel is metabolised by cytochrome P450 in the liver, where it is first transformed into 2-oxo-clopidogrel, an intermediate metabolite, by CYP2C19, CYP1A2, and CYP2B6. Subsequently, 2-oxo-clopidogrel is transformed into an active thiol metabolite, which is catalysed by the CYP2C9, CYP3A4, CYP2B6 and CYP2C19 isoforms and the paraoxonase (PON)-1 enzyme. The active metabolites irreversibly bind to the P2RY12 on the platelet membrane to inhibit ADP, preventing the binding of fibrinogen to the GPIIb/IIIa receptor and ADP-mediated activation of the GPIIb/IIIa complex, thus preventing platelet activation and exerting antiplatelet effects (Fig. 1)^6,7^.

Third-generation thienopyridine prasugrel exhibited better pharmacokinetic properties than clopidogrel. Following intestinal absorption of prasugrel, which is regulated by P-gp, prasugrel is metabolised by intestinal esterification to the intermediate metabolite thiolactone. This requires only one hepatic metabolic step to produce active metabolites, primarily by CYP3A4 and CYP2B6, with minor contributions from CYP2C19 and CYP2C94. Cyclopentyltriazolopyrimidine (CPTP) ticagrelor is a non-thienopyridine quickly absorbed in the intestines. CPTP acts directly on P2RY12 through reversible binding and does not require additional biotransformation for activation^4^.

#### Genetic polymorphisms affecting Clopidogrel response

Despite being one of the most widely used antiplatelet medications, clopidogrel has variable effects in certain patient populations. Genetic polymorphisms in *CYP2C19* are among the main variables that influence an individual’s response to clopidogrel^8^. CYP2C19 plays a pivotal role in the formation of active metabolites, being involved in both metabolic steps as illustrated in Fig. 1. However, *CYP2C19* gene polymorphisms are common, with more than 25 alleles identified, each conferring varying degrees of enzymatic activity and contributing to interpatient variability in antiplatelet effects^9^.

Among loss-of-function (LOF) alleles, CYP2C19*2 and *3 are the most prevalent and widely investigated. Homozygous or heterozygous patients are categorised as poor or intermediate metabolizers, respectively, because of their almost absent or reduced enzymatic activity^9^. Ellithi et al. reported that *CYP2C19* LOF carriers exhibited a consistent reduction in active clopidogrel metabolite levels, increased platelet reactivity, and increased risk of ischaemic events^10^. Previous studies have suggested that the risk of clopidogrel resistance increases in the presence of the CYP2C19*2 and *3 polymorphic alleles^11,12^. Moreover, these alleles may be independent predictors of elevated platelet reactivity in patients treated with clopidogrel^13^. The presence of CYP2C19*2 and *3 alleles may be considered independent risk factors for stroke recurrence in patients with ischaemic stroke^14^. Furthermore, research findings indicate that patients with LOF of *CYP2C19* exhibit a higher frequency of ischaemic events and an increased risk of stent thrombosis^15^. Two meta-analyses of clopidogrel-treated patients revealed that *CYP2C19* LOF allele carriers were more likely to experience stroke and other combined vascular events than were noncarriers^16,17^. This finding validates the increased risk of clopidogrel failure in the prevention of cardiovascular events in patients with *CYP2C19* LOF variants^16,17^.

The CYP2C19*17 allele has been linked to increased enzyme activity, and depending on the number of alleles present, it is associated with “rapid” metabolism (1 *17 allele) and 'ultra-rapid' metabolism (2 *17 alleles)^9^. CYP2C19*17 is strongly associated with an enhanced response to clopidogrel and a higher risk of bleeding^18,19^. Another study did not show a significant link between CYP2C19*17 and platelet inhibition or the risk of high platelet response^13^. In real-world genotype-guided antiplatelet therapy, the CYP2C19*17 allele was not significantly associated with post-PCI prescription decisions or clinical outcomes^20^. These findings suggest that the *CYP2C19* *1/*17 and *17/*17 genotypes have limited clinical utility in guiding antiplatelet therapy after PCI^20^.

Numerous studies have identified significant interethnic variability in *CYP2C19* polymorphisms. The most prevalent allele in Asian populations is CYP2C19*2, and its frequency varies by region. The frequency of the CYP2C19*2 allele in the Iranian population was 21.4%^21^, which is higher than that in Swedish (14.4%)^22^, German (15%)^23^, Ethiopian (13.6%)^24^, and Zimbabwean (13.1%)^25^ populations but lower than that in Japanese (23%)^26^ and Chinese-Taiwanese (32%)^26^ populations. The Filipino population had the highest frequency of the CYP2C19*2 allele (39%)^26^. However, the prevalence of the CYP2C19*2 allele has noticeably increased from Iran and Western Asia toward India, reaching its highest frequency, over 75%, in Melanesian populations^27^. Additionally, the CYP2C19*3 allele was detected with a prevalence of 1.7% in the Iranian population^21^ but was absent in the Canadian^28^ and Danish^29^ populations. However, the CYP2C19*3 allele was more frequent in other populations such as the Japanese (10.4%)^26^ and Korean (11.6%)^30^, whereas it was comparable to that reported in the Ethiopian population (1.8%)^31^. The prevalence of the CYP2C19*3 variant, commonly referred to as an Asian mutation^32^, has increased progressively from the western to eastern regions of Asia. Previous research has identified the highest frequency of the CYP2C19*3 allele in Southeast Asian populations, with 37% reported in Indonesians and 34% in the Iruna population of New Guinea^33^. Moreover, the frequency of the CYP2C19*17 allele in the Iranian population was 27.1%^21^ greater than that in other populations, including Denmark (20.1%)^31^, Norway (22%)^34^, African-Americans (21%)^35^, Ethiopia (17.9%)^36^, Korea (1.5%)^37^ and Japan (1.3%)^38^. This roughly resembles the frequency recorded for the Polish population (27.2%)^39^. Therefore, assessing the allele and genotype frequencies of CYP2C19 across various ethnic groups offers valuable insights for tailoring treatments to enhance therapeutic efficacy, minimise adverse effects, and support the progression of personalised medicine^21^.

Current guidelines and expert consensus recommend routine genetic testing in patients receiving clopidogrel^20,40^. According to evidence-based recommendations of the Clinical Pharmacogenetics Implementation Consortium (CPIC), the administration of clopidogrel to individuals with a poor or intermediate CYP2C19 metabolizer phenotype should be avoided. Instead, these patients should be administered an alternative P2Y12 inhibitor such as ticagrelor or prasugrel. However, clopidogrel can be considered for individuals with a normal, rapid, or ultra-rapid CYP2C19 metabolizer phenotype^20,40^. Recent meta-analyses have shown that patients undergoing PCI who benefit the most from alternative antiplatelet therapy may be identified using *CYP2C19* genotype-guided therapy^4,41^. Clopidogrel is still commonly prescribed for ACS and non-ACS patients undergoing PCI, as well as for patients with other cardiovascular and neurovascular indications or who are at increased risk for bleeding^20^. Although newer and more potent P2Y12 inhibitors, such as prasugrel and ticagrelor, have demonstrated superior efficacy in reducing ischaemic events compared with clopidogrel in randomised trials involving patients with ACS, they are frequently associated with a higher risk of bleeding complications. Real-world studies involving patients with ACS have not consistently demonstrated that newer P2Y12 inhibitors outperform clopidogrel in terms of their clinical effectiveness. Clopidogrel remains a cornerstone of antiplatelet therapy, supported by robust evidence across various clinical contexts, underscoring its critical role in routine medical practice^42^.

A precision medicine strategy that used *CYP2C19* genetic test results to deliver ticagrelor or prasugrel to LOF carriers and clopidogrel to non-carriers provided a more well-rounded therapeutic approach, as it lowered the risk of bleeding and ischaemic events compared to the universal use of ticagrelor or prasugrel^43^. Therefore, current evidence supports the use of *CYP2C19* genetic testing before prescribing oral P2Y12 inhibitors in patients with ACS or those undergoing PCI^43^.

There is conflicting information regarding polymorphisms in *ABCB1*, which encodes a P-gp transporter and is involved in the efflux process of several medications, including clopidogrel^44^. Approximately 50 single-nucleotide polymorphisms (SNPs) are found in *ABCB1*; the most studied is C3435T, which is linked to P-gp^7^. Variations in P-gp expression between individuals caused by polymorphisms in the *ABCB1* gene could change clopidogrel absorption in vivo and the quantity of parent medication reaching the liver^45^. The 3435 T allele may be connected to clopidogrel hyporesponsiveness, as one study on the *ABCB1* C3435T gene polymorphism revealed that patients with the TT and CT genotypes had decreased clopidogrel absorption and an increased risk of adverse cardiovascular events compared to patients with the CC genotype^46^. These findings are in line with those of a study on patients with coronary artery disease (CAD) and ACS, which revealed that patients with the TT genotype had a greater risk of major adverse cardiovascular events (MACEs) than those with the CC or CT genotype^47^. Nevertheless, some recent studies have failed to discover a relationship between the effectiveness of clopidogrel and *ABCB1* gene polymorphisms^48^, suggesting that the *ABCB1* genotype has no effect on platelet reactivity in patients receiving clopidogrel^49^. Furthermore, the risk of clopidogrel resistance is not strongly correlated with the presence of the *ABCB1* mutant allele^12,50^. It is still highly debatable whether the *ABCB1* C3435T gene polymorphism influences the antiplatelet effect of clopidogrel and whether this polymorphism can predict high platelet reactivity following clopidogrel treatment. In contrast, the TT genotype may or may not be a factor in modifying treatment plans for patients who do not respond well to clopidogrel^51^. There is currently no clinical role for testing these polymorphisms in patients treated with clopidogrel, as the test has not demonstrated clinical utility at this time.

CES1 plays an essential role in clopidogrel biotransformation. CES1 hydrolyses 85% of the prodrug clopidogrel to an inactive carboxylic acid derivative. Thus, cytochrome P450 enzymes ultimately convert a small amount of clopidogrel into its active metabolites. Changes in enzyme activity can result from mutations in *CES1*, which encodes members of the carboxylesterase family that are important hepatic enzymes for drug clearance^52^. Variations in the *CES1* gene result in changes in the activity of the CES1 enzyme, which is negatively correlated with the generation of active metabolites of clopidogrel and impacts the antiplatelet effects of this drug^7,19,53^. According to Gao et al., the *CES1* G143E (rs71647871) polymorphism is anticipated to be a predictor of platelet hyporesponsiveness to clopidogrel because this mutation results in reduced enzyme catalytic activity^51^. Mirzaev et al. reported that the *CES1* rs2244613 polymorphism is associated with an increased risk of clopidogrel resistance, elevated platelet reactivity, and increased enzymatic activity^54^. Nevertheless, other studies have demonstrated that the *CES1* rs2244613 SNP is not associated with the risk of MACEs and has no effect on the effectiveness of clopidogrel in inhibiting platelet aggregation^55^. However, further research is needed to determine the precise influence and correlation of *CES1* rs2244613 SNP with high platelet reactivity, given the absence of related studies and inconsistent results^51^. At present, testing for these polymorphisms in patients receiving clopidogrel is not clinically indicated, as there is no established evidence supporting its clinical usefulness.

The key enzyme in the second step of clopidogrel metabolism is paraoxonase-1 (PON1). Based on in vitro findings, PON1 is the rate-limiting enzyme in the formation of the active thiol metabolite of clopidogrel^56^. A study conducted by Bouman et al. revealed an association between the presence of *PON1* QQ192 and a significant decrease in PON1 activity, leading to decreased levels of the active metabolite clopidogrel and an increased risk of stent thrombosis^56^. Subsequently, another study on patients with or without ACS revealed that the *PON1* Q-allele was strongly linked to poor cardiovascular outcomes but not to the antiplatelet effect of clopidogrel^57^. In contrast, the *PON1* 192R variants were found to reduce the impact of clopidogrel on platelet aggregation. The impact of the *PON1* 192Q allele on platelet function and clopidogrel efficacy is considered minimal, suggesting that the *PON1* 192R variant may independently increase the risk of high platelet reactivity^13^. A study examining the impact of *PON1* polymorphisms on MACEs and clopidogrel efficacy revealed that patients with the RR genotype had a greater risk of clopidogrel resistance; however, no association with clinical outcomes was detected^58^. Furthermore, in patients with ACS, the RR genotype is a risk predictor for PCI followed by revascularization^59^. However, a recent study showed that the *PON1* Q192R polymorphism had no effect on clopidogrel pharmacokinetics and antiplatelet activity^60^. Thus, there is still a discrepancy regarding which allele, Q or R, interferes with the effect of the *PON1* Q192R polymorphism on the response to clopidogrel.

Previous research on *P2RY12* has mainly focused on T744C, C34T, and G52T variants. The *P2RY12* 744T allele is associated with an increased risk of MACEs and high on-treatment platelet reactivity after PCI^61^. A meta-analysis did not reveal any impact of the *P2RY12* T744C polymorphism on clopidogrel resistance or high on-treatment platelet reactivity^62^. Similar results have been reported by Nie et al. ^63^. Two recent observational studies of patients undergoing percutaneous neurointervention revealed no correlation between high platelet reactivity during treatment and the T744C allele^64^. A study of Chinese patients undergoing PCI revealed a correlation between genetic variations in C34T and G52T and reduced clopidogrel responsiveness and adverse clinical events^65^. Furthermore, a meta-analysis revealed that patients on clopidogrel may be at risk for increased platelet reactivity owing to the presence of the T alleles of C34T and G52T^61^. Interestingly, another meta-analysis revealed that G52T and C34T variations in the *P2RY12* gene were associated with cardiovascular events in the Chinese population, but not in the Caucasian population^66^.

Research has indicated inconsistencies in the findings of ongoing investigations on the effect of *P2RY12* T744C on clopidogrel effectiveness. Studies on C34T and G52T have produced encouraging results but are not direct enough to guide clinical care. *P2RY12* gene correlation studies offer research ideas for predicting platelet reactivity following clopidogrel treatment, but there are insufficient data to justify the clinical implementation of these ideas. The effects of genetic polymorphisms on clopidogrel response are summarised in Table 1.

**Table 1:** Genetic polymorphisms and their impact on clopidogrel response

Polymorphism	Impact on clopidogrel response	Population	Influence on clinical outcome
CYP2C19*2 and *3	Increased clopidogrel resistance	Chinese patients^11,12^	Stroke recurrence^14^
			Risk of stent thrombosis^15^
			Cardiovascular events^16,17^
CYP2C19*17	Strongly linked to an enhanced response to clopidogrel	German patients^18^ and Caucasian ICPC subjects^19^	High risk of bleeding^18,19^
	No significant association was shown with platelet inhibition or risk of HPR	Chinese patients^20^	No effect on post-PCI^20^
*ABCB1* (C3435T)	Connected to clopidogrel hyporesponsiveness	Chinese patients^46^	MACEs^46,47^
	Not strongly associated with clopidogrel resistance It is still highly debatable whether the *ABCB1* C3435T gene polymorphism influences the antiplatelet effect of clopidogrel	Croatian^48^, Chinese^12,49^ and Iranian Patients^50^	NA
*CES1* G143E (rs71647871)	Associated with higher plasma concentrations of clopidogrel-active metabolite, and hence may enhance antiplatelet activity	Chinese patients^52^	NA
*CES1* (rs2244613)	Increased the level of residual platelet reactivity and risk of clopidogrel resistance	Russian patients^54^	NA
	No effect on the effectiveness of clopidogrel There was still a lack of relevant studies and inconsistent results	In Egyptians^55^	Not associated with the risk of MACEs^55^
*PON1* 192Q	Decreased levels of the active metabolite clopidogrel	In the European population^56^	Stent thrombosis^56^
	No effect on platelet function and clopidogrel efficacy	In Chinese patients^13^ and Korean patients^57^	Strongly linked to poor cardiovascular outcomes^57^
*PON1* 192 R	High risk of clopidogrel resistance	In Chinese patients^13,58^	No association with clinical outcomes was detected^58^
			PCI^59^
	There is no effect on the antiplatelet or pharmacokinetic properties of clopidogrel. It is still unclear which allele, Q or R, is associated with CR	In TRITON-TIMI 38 trial^60^	NA
*P2RY12* T744C	Effect on clopidogrel resistance or high on-treatment platelet reactivity	In Chinese patients^61^	MACEs and PCI^61^
	No correlation with on clopidogrel resistance and high platelet reactivity. Research indicates inconsistencies in the findings	A meta-analysis study was conducted in French, Americans, Chinese, Croatian, Egyptian, and the Czech Republic populations^62^. Chinese patients^63,64^	NA
*P2RY12* C34T and G52T	Reduced clopidogrel responsiveness. Results were not direct enough to guide clinical care	In Chinese patients^61,65^	Cardiovascular events^66^

*ICPC* international classification of primary care, *HPR* high platelet response, *MACEs* major adverse cardiovascular events, *NA* not available.

### The pharmacogenetics of aspirin

Acetylsalicylic acid (ASA), also referred to as aspirin, is a vital medication that is frequently used as antiplatelet therapy to prevent recurrent ischaemic events in patients with ischaemic stroke^67^. Clopidogrel combined with aspirin is a well-established, commonly used dual antiplatelet therapy for individuals with ischaemic heart disease to reduce ischaemic risk^68^.

#### The therapeutic targets of aspirin

**Fig. 2:**
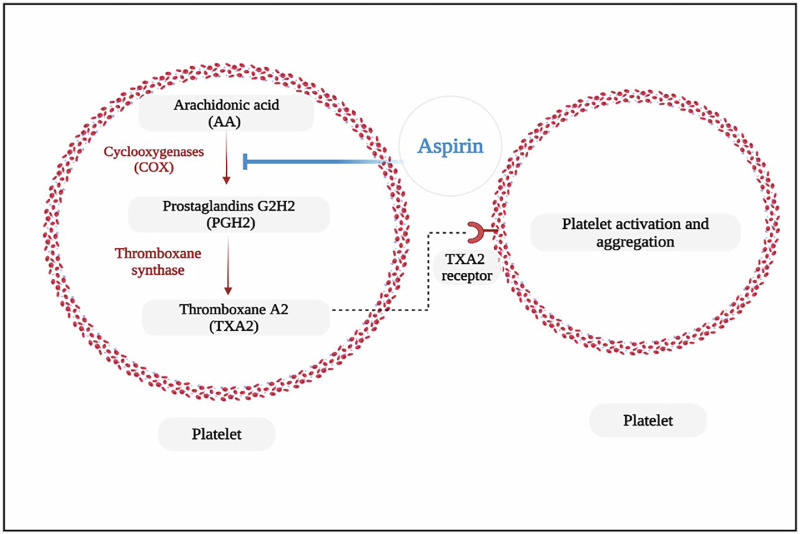
Aspirin antiplatelet effect pathway. Aspirin is an inhibitor of cyclooxygenase (COX) that catalyses the transformation of arachidonic acid (AA) into prostaglandins G2 and H2. Thromboxane A2 (TXA2) is formed by thromboxane synthase and binds to the TXA2 receptors of nearby platelets, thereby serving as a potent platelet activator (modified from ref. ^69^). Figure created with Bio Render.com.

The therapeutic target of this drug is to inhibit the function of prostaglandin G/H synthases 1 and 2 (PTGS1 and PTGS2), which are also referred to as cyclooxygenases (COX)-1 and -2, respectively (Fig. 2). COX-2 is highly inducible, primarily by inflammation, whereas COX-1 is constitutively produced. Mature platelets express the COX-1 enzyme, which catalyses the transformation of arachidonic acid (AA) into prostaglandins G2 and H2, producing thromboxane A2 (TXA2). When TXA2 is released into the bloodstream, it binds to TXA2 receptors on nearby platelets and activates them. Furthermore, TXA2 collaborates with other chemicals released by activated platelets such as fibrinogen, adenosine diphosphate (ADP), and factor V to accelerate the process^69^.

#### Genetic polymorphisms affecting aspirin response

Genetic variations that alter drug metabolism, leading to aspirin resistance (AR), are frequently reported phenomena that can cause treatment failure in patients, even in those who experience the well-established benefits of ASA^70^. Two genes, *PTGS1* and *PTGS2*, encode COX-1 and COX-2, which are enzymes that catalyse the conversion of AA to prostaglandins and thromboxane^69^. Both *PTGS1* and *PTGS2* have been studied extensively. The C allele and CC genotype of the rs1330344 variant of the *PTGS1* gene encoding COX-1 are linked to AR and increased levels of AA-induced aggregation^69^. A similar finding was noted by Li et al. ^71^. In Chinese stroke patients receiving aspirin therapy, the CC genotype has been linked to poor functional outcomes^72^. The C allele has also been linked to a higher risk of ischaemic stroke in the Chinese population^73^. Nevertheless, the outcomes have not been consistent, and this polymorphism may have increased COX-1 activity and did not affect the platelet response to aspirin^74,75^. Additionally, rs10306114, another SNP in *PTGS1*, did not correlate with AR^69^. Although *PTGS2* is a highly polymorphic gene, its expression and function are affected only by a few SNPs^76,77^. Recently, in patients with ischaemic stroke carrying the GC or CC genotype, the rs20417 C allele was found to be strongly correlated with AR. Importantly, not all studies corroborated or validated these findings^78^.

The specific TXA2 receptor (TBXA2R) is encoded by the *TBXA2R* gene, and variations in this gene can affect ADP-induced platelet aggregation. The *TBXA2R* polymorphism rs4523 has been linked to platelet aggregation in patients with CAD who receive aspirin. Patients with the TT and CT genotypes showed greater platelet aggregation than those with the CC genotype^69^. In previous investigations, TT homozygotes also demonstrated elevated platelet reactivity related to AR^79^. The other SNP (rs1131882) of the *TBXA2R* gene is not associated with AR^69,80^.

The platelet membrane receptors P2Y1 and P2Y12 are essential for platelet aggregation, thrombosis and antiplatelet medication pharmacology^81^. While the inhibition of *P2RY12* inhibits ADP-induced platelet activation, ADP amplifies multiple signalling pathways to activate platelets through autocrine and paracrine mechanisms^82^. Goodman et al. reported no correlation between these polymorphisms and AR^83^.

Genetic variations in platelet endothelial aggregation receptor 1 (PEAR1) were previously thought to be linked to platelet aggregation and the response to aspirin^84^. However, the relationship between these findings and cardiovascular clinical outcomes remains unclear. PEAR1 encodes a type 1 membrane protein that is expressed in endothelial cells and platelets. Consequently, a clinical outcome analysis of aspirin and the *PEAR1* rs12041331 G > A variant has recently been published^19^. The authors examined bleeding, cardiovascular incidents, and cerebrovascular events; however, none of these clinical outcomes was significantly associated with this *PEAR1* variant. However, the effects of *PEAR1 rs12041331* might vary depending on the type of stroke and whether aspirin is being administered for primary or secondary stroke prevention. According to a recently published pharmacogenetic analysis by Li et al., *PEAR1* rs12041331 was significantly associated with functional outcomes only in patients with the small-artery occlusion subtype of stroke treated with aspirin alone^85^. Thus, the reason for the discrepancy in the results for the *PEAR1* rs12041331 variant between the studies could be the difference in disease severity among patients. Thus, it is possible that *PEAR1* rs12041331 interacts with aspirin only in patients with specific stroke subtypes. Further research is required to confirm these results.

Platelet adhesion and aggregation control depend on glycoprotein IIb/IIIa (GPIIb/IIIa)^86^. Different levels of aspirin sensitivity have been associated with variations in the integrin subunit beta 3 (*ITGB3*) gene, which encodes the fibrinogen glycoprotein GPIIb/IIIa. Szczeklik et al. found that PlA1/A2 allele carriers had higher AR than wild-type carriers^87^. According to a meta-analysis by Goodman et al., AR measured using platelet function assays was not associated with *PlA1/A2* polymorphisms in patients with CVD^83^. Another meta-analysis confirmed the relationship between the single-nucleotide substitution rs5918 in the *ITGB3* gene of GPIIIa and clinical outcomes in patients receiving aspirin, showing no significant association with MACEs^88^. Other studies have reported contradictory results, with no significant association between glycoprotein IIIa P1A1/A2 polymorphism and AR or stroke development^89,90^.

α-2A-adrenergic receptor (*ADRA2A*) gene polymorphism is associated with AA-induced aggregation. *ADRA2A* encodes α-2A-adrenergic receptor, which is involved in epinephrine-induced platelet aggregation. Greater levels of AA-induced aggregation were linked to the minor allele T (rs4311994) of *ADRA2A*. This allele has been linked to increased platelet reactivity to aspirin in individuals with type 2 diabetes^91^, but these outcomes are not always repeatable^92^.

In addition to the efficacy studies mentioned earlier, several other recent studies have assessed the correlation between genetic variants and side effects of aspirin on the gastrointestinal tract. Researchers have discovered a variant in the intron of *EYA1* with a statistically significant association with the side effects of aspirin in the gastrointestinal tract, raising the possibility of aspirin-induced peptic ulcer disease. Lower *EYA1* expression in the epithelium of the upper gastrointestinal tract may alter this risk; however, further research is needed to determine the functional underpinnings of this relationship^93^. According to recently published candidate gene association studies, *PTGS1*, *NOS3*, *CHST2*, and *GSTP1* variants have been linked to gastrointestinal bleeding in aspirin-treated patients^94–97^.

Genetic testing holds significant value in personalised medicine, as tailoring treatment strategies for AR patients based on genetic polymorphisms may reduce treatment failures and improve clinical outcomes, although further studies are imperative^98^. The effect of genetic polymorphisms on aspirin response is summarised in Table 2.

**Table 2:** Genetic polymorphisms and their impact on aspirin response

Polymorphism	Impact on Aspirin response	Population	Influences on clinical outcome
*PTGS1* CC (rs1330344)	Linked to AR	Caucasian^69^ and Chinese patients^71^	Stroke^73^
	No correlation with AR	Chinese^74^ and Italian patients^75^	NA
*PTGS1* (rs10306114)	Did not exhibit any correlation with AR	Caucasian patients^69^	NA
*PTGS2* GC or CC (rs20417)	Strongly correlated with AR Not all related research corroborates or validates these findings	Chinese patients^78^	NA
*TBXA2R* the TT and CT (rs4523)	Linked AR to platelet aggregation	Caucasian^69^ and Chinese patients^79^	NA
*TBXA2R* rs1131882	Did not associated with AR	Caucasian^69^ and Chinese patients^80^	NA
*P2RY1* and *P2RY12*	No association between these polymorphisms and AR	A comprehensive systematic review^83^	NA
*PEAR1* (rs12041331)	Linked to platelet aggregation and aspirin response. Further research is needed to confirm these explanations	White and African-American populations^84^	Not associated with bleeding, cardiovascular events, or cerebrovascular events^19^
			The impact of this SNP on aspirin depends on the stroke subtype^85^
*ITGB3* PlA1/A2	PIA2 polymorphism reduces the effectiveness of aspirin’s antithrombotic effects	Polish population^87^	Not associated with CVD^83^
			No significant association with MACE^88^
	No significant correlation with AR	Tunisian patients^90^	No significant association with stroke development^89,90^
*ADRA2A* (rs4311994)	Linked to increased platelet reactivity to aspirin	Caucasian patients^91^	NA
*EYA1*	Association with aspirin side effects	From UK hospitals^93^	Gastrointestinal tract^93^
*PTGS1, NOS3, CHST2*, and *GSTP1*	Association with aspirin side effects	Spainish^94,95^, Brazilian^96^ and Japanese populations^97^	Gastrointestinal bleeding^94–97^

*AR* aspirin resistance, *CVD* cardiovascular disease, *MACEs* major adverse cardiovascular events, *NA* not available.

## The pharmacogenetics of anticoagulants

### The pharmacogenetics of warfarin

**Fig. 3:**
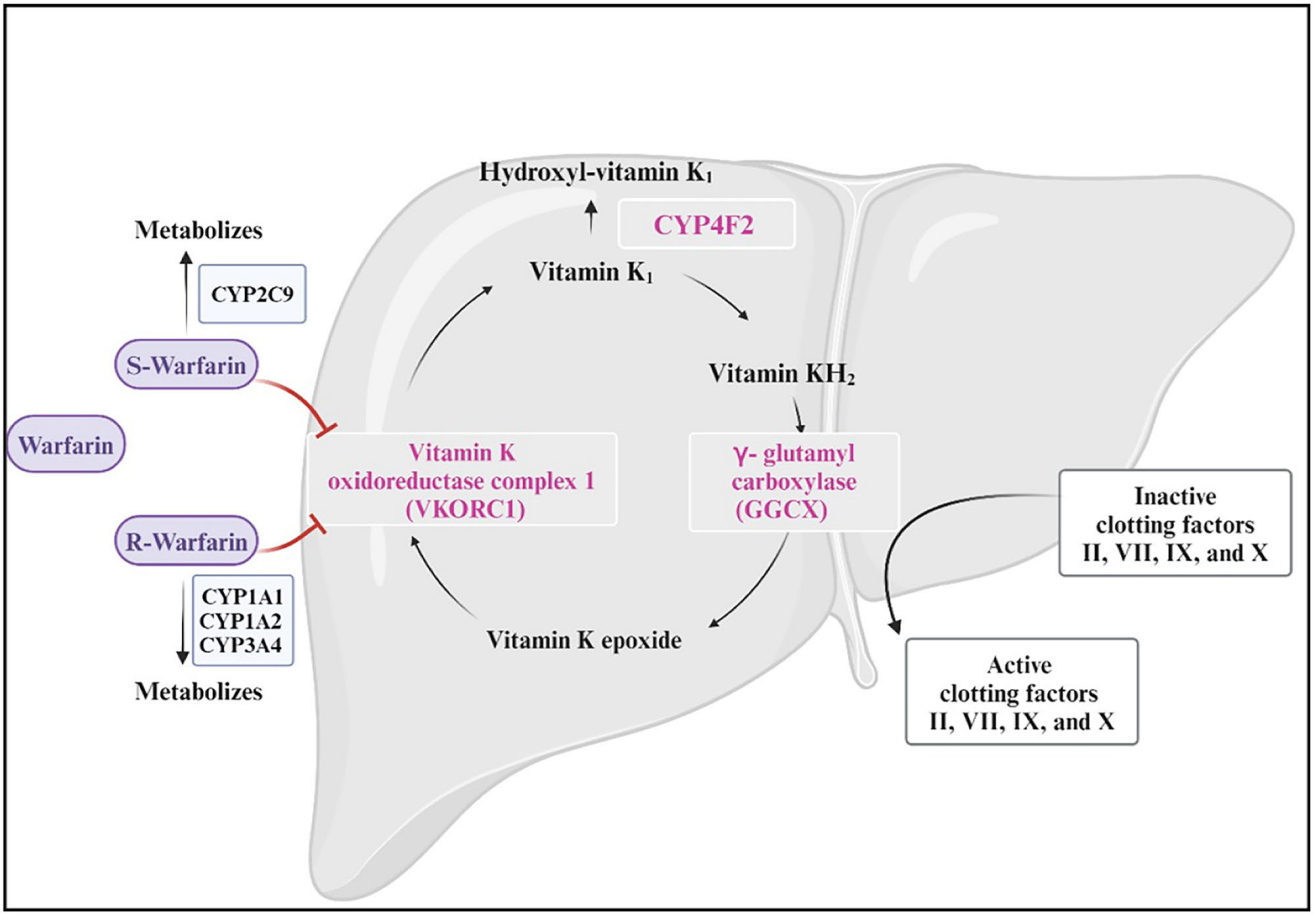
Mechanism of action of warfarin. Warfarin inhibits the formation of the active form of vitamin K. Warfarin—clinically available as an equal mixture of R and S enantiomers—inhibits vitamin K oxidoreductase complex 1 (VKORC1) and, therefore, prevents the reduction of vitamin K_1_ to vitamin KH2. KH2 is necessary for γ-glutamyl carboxylase (GGCX) expression and activation of clotting factors II, VII, IX, and X (modified from ref. ^101^). Figure created with Bio Render.com.

Globally, warfarin is the most frequently prescribed oral anticoagulant for treating and preventing thromboembolic disorders and preventing stroke in patients with atrial fibrillation (AF)^99^. Warfarin is given as a racemic mixture of its R and S isomers. The S isomer is three to five times stronger than the R isomer and is metabolised via a different pathway. CYP2C9 is the main enzyme involved in S-warfarin metabolic clearance, whereas CYP1A1, CYP1A2 and CYP3A4 are among the cytochrome P450 enzymes that remove R-warfarin (Fig. 3)^100^.

#### The mechanism of action of warfarin

The mechanism by which warfarin produces its anticoagulant therapeutic effect is by inhibiting the enzyme vitamin K oxidoreductase complex 1 (VKORC1). This enzyme converts vitamin K epoxide to vitamin K_1_, which is subsequently converted to vitamin KH_2_. Vitamin KH_2_ is an important cofactor for the enzyme γ-glutamyl carboxylase (GGCX), which activates clotting factors II, VII, IX, and X, which are essential for blood clotting. Vitamin K_1_ is metabolised by the CYP4F2 enzyme to hydroxyl-vitamin K_1_, an inactive metabolite, to reduce the amount of vitamin K_1_ that can be reduced to vitamin KH_2_^101^. A narrow therapeutic index and wide inter-individual variation in dose requirements and response to treatment pose challenges to warfarin treatment and increase the risk of treatment complications. As a result, regular monitoring of the international normalised ratio (INR) is necessary to guarantee optimal anticoagulation and lower the risk of either a low or high INR (which carries the risk of thrombosis or bleeding, respectively)^101,102^.

#### Genetic polymorphisms affecting warfarin response

Genetic polymorphisms significantly influence dose requirements, yet they are infrequently incorporated into clinical practice. Warfarin was the first medication to have established pharmacogenetic dosing guidelines^101^. According to the 2017 CPIC guidelines, genotype-guided warfarin therapy is based on variations in *CYP2C9*, *VKORC1*, *CYP4F2* and rs12777823. These guidelines use validated pharmacogenetic algorithms to determine appropriate dosing.

Patients with *CYP2C9* and *VKORC1* polymorphisms may require adjusted starting doses to achieve the intended anticoagulant effects because these variations contribute to reduced warfarin metabolism and altered sensitivity^101,103^. The effectiveness of a genotype-guided warfarin dosing strategy compared to either a fixed (e.g. 5 mg/day) or clinically guided dosing strategy has been investigated in three large randomised controlled trials: COAG, EU-PACT, and GIFT. In the EU-PACT and GIFT clinical trials, over 90% of the patients in the COAG group were of European ancestry, whereas 27% were of European ancestry. EU-PACT and GIFT demonstrated improved outcomes, while COAG found no distinction between genotype-guided and clinically guided dosing approaches^104^.

The primary metabolizer of S-warfarin and several other commonly prescribed drugs is CYP2C9, an enzyme belonging to the CYP2C subfamily of cytochrome P450 enzymes^105^. Studies have shown that CYP2C9*2, *3, *5, *6, *8 and *11 are linked to decreased function of the CYP2C9 enzyme, which lowers the clearance of S-warfarin, the more active enantiomer, and consequently lowers patient dosage requirements. Individuals with these genotypes are more likely to experience severe bleeding complications and thus require lower doses. The most prevalent alleles in individuals of European ancestry are *2 and *3, whereas those in individuals of African ancestry are primarily the *5, *6, *8 and *11 alleles^101,103,104^.

The target enzyme of warfarin, vitamin K epoxide reductase protein, is encoded by the *VKORC1* gene. The rate-limiting step in vitamin K recycling is the conversion of vitamin K epoxide to vitamin K, which is catalysed by VKORC1. SNPs in this gene contribute to warfarin resistance, and extremely high doses of medication are required for therapeutic anticoagulation. A common variant in the *VKORC1* regulatory region, c.1639 G > A (rs9923231), is associated with decreased VKORC1 expression and patients with this variant require lower warfarin doses. Asians are more likely to carry the -1639 AA (susceptible) genotype than individuals of European descent, whereas African-Americans are more likely to carry the -1639 GG (reduced sensitivity) genotype. This difference explains the higher reported doses of warfarin required in Africans compared to Europeans and the frequently lower reported doses required in Asians^101,103^.

As a counterpart to VKORC1, CYP4F2 prevents vitamin K from building up by converting it to hydroxyl-vitamin K_1_ and eliminating it from the vitamin K cycle. Enzyme activity is affected by the presence of the *CYP4F*2*3 variant, which leads to increased availability of vitamin K_1_ for the reduction of vitamin K hydroquinone and activation of clotting factors. In the Asian and European cohorts, the *3 allele was associated with higher warfarin doses than the *1 allele. No association was found among the populations of African descent^101,103^. The Food and Drug Administration (FDA) has approved warfarin-labelling information, but does not currently list *CYP4F2* as a biomarker. However, in addition to testing for *CYP2C9* and *VKORC1* variants, some clinical laboratories may test for the *CYP4F*2*3 allele. The 2017 CPIC warfarin dosing guidelines include recommendations based on the *CYP4F2* genotype when results are available^103^. Another variant, rs12777823, located close to the *CYP2C* cluster on chromosome 10, is associated with reduced warfarin dosage requirements. Reduced S-warfarin clearance was also associated with the SNP rs12777823. This SNP is prevalent in the general population but appears to be specific to African-Americans regarding its relationship with warfarin response^101^.

Mal, T Cell Differentiation Protein Like (*MALL*) gene has recently been identified as a factor that could indirectly influence warfarin response through its interactions with caveolin-1^106^. The integrity of endothelial cells, which is responsible for controlling the haemostatic pathway^107^, is regulated by the caveolin-1 membrane protein^108^. As the *MALL*-encoded protein interacts with caveolin-1, the indirect influence of *MALL* on the anticoagulant action of warfarin is biologically plausible. *MALL* has been reported to influence warfarin dosage solely in patients of African descent, although direct evidence of biological plausibility is still required^106^. A recent study emphasised the necessity for larger pharmacogenomic studies of warfarin in patients of African descent to identify genetic variants specific to this group and ultimately enhance the quality of anticoagulant therapy for this under-researched population^106^. The use of genetic information to guide warfarin dosing in clinical practice has been influenced by ethnic group variations due to genetic differences that affect drug metabolism and response. Variations in *CYP2C9* result in slower warfarin metabolism, which increases the risk of bleeding. CYP2C9*2 and *3 genetic variants were more common in European populations than in other ethnic groups, whereas the *5, *6, *8, and *11 alleles were more frequent among African populations. Individuals with these variations usually need lower dosages of warfarin^101,103,104^. *VKORC1* polymorphisms are more prevalent in Asian and African ethnicities and are associated with a higher sensitivity to warfarin, making these individuals require lower doses^101,103^. Because of the impact of the CYP4F2* 3 variant on enzyme activity, clotting factors are activated, which increases the risk of clotting. The prevalence of CYP4F2*3 variation was higher in Asian and European cohorts than in African cohorts, and higher doses of warfarin were needed^101,103^. Another variant, rs12777823, which is close to *CYP2C*, has been linked to decreased S-warfarin clearance. This SNP is specific to African-Americans and is required to reduce the warfarin dosage^101^. The recently discovered MALL locus may have an indirect impact on warfarin response, particularly in African-American patients. Although it has been associated with warfarin dosage requirements, further research is needed to confirm its biological plausibility^106^.

## The pharmacogenetics of lipid-lowering drugs

### The pharmacogenetics of hydroxymethylglutaryl coenzyme A reductase inhibitors

Statins, also called 3-hydroxy-3-methylglutaryl coenzyme A (HMG-CoA) reductase inhibitors, are the first-line therapy for lowering lipid levels and reducing CVD risk^109–111^.

#### The therapeutic target of statins

Statins exert their therapeutic effects by inhibiting the active site of HMG-CoA reductase, the rate-limiting enzyme in cholesterol synthesis, resulting in lower levels of low-density lipoprotein cholesterol (LDL-C) in the blood. The conversion of HMG-CoA to mevalonic acid is inhibited by preventing substrate access to this site, which leads to reduced hepatic cholesterol synthesis and upregulation of LDL receptors (LDLRs). This further leads to a decrease in the amount of LDL-C in the blood and an increase in LDL-C removal from circulation^112^. However, not every patient responds well to statins, and some do not achieve their target cholesterol reduction levels. Moreover, a large proportion of patients experience side effects. Statin-induced myopathy (SIM) and statin-associated muscle symptoms (SAMs) are the most frequently reported side effects of statins, and these side effects result in poor adherence to or cessation of statin pharmacotherapy regimens^113,114^. Many genes have been studied to determine their potential influence on various interactions of statins. Genes involved in absorption, distribution, and metabolism are associated with fat metabolism, and these differences can be affected^115^.

#### Genetic polymorphisms affecting statin response

Liver cells contain a protein known as organic anion transporting polypeptide 1B1 (OATP1B1), encoded by the solute carrier organic anion transporter family member 1B1 (*SLCO1B1*) gene. Toxins, medications, hormones, and bilirubin are all transported by OATP1B1 into the liver, where they are eliminated from the body. The OATP1B1 protein typically transports several medications, including statins (such as rosuvastatin and pitavastatin)^109^. Pitavastatin and rosuvastatin have been suggested by Prueksaritanont et al. as probe substrates for OATP1B, and they have demonstrated relative sensitivity and selectivity to OATP1B inhibitors. Pitavastatin was meant to be a more selective and sensitive clinical OATP1B probe than rosuvastatin^116^. The effect of the missense variant rs4149056 in *SLCO1B1*, which causes impaired hepatic clearance and elevated plasma statin concentrations, has been studied^117^. When simvastatin was used, a clear correlation was observed between increased exposure to simvastatin and a greater risk of developing myopathy^118^. The *SLCO1B1* SNP has been shown to decrease the OATP1B1 transporter activity^119^. As a result, rosuvastatin concentration and plasma exposure were elevated, increasing the risk of SIM^117^.

The ATP-binding cassette G2 (ABCG2) protein, also referred to as breast cancer resistance protein (BCRP), functions as an intermediary for the cellular outflow of various exogenous substances, such as antibiotics, chemotherapeutic agents, and dietary toxins, as well as endogenous substances such as oestrogens^120^. In tumour cells, ABCG2 causes resistance to several anticancer medications; however, in normal cells, ABCG2 appears to protect cells against cytotoxic substances^120^. Rosuvastatin is actively taken up into hepatocytes from portal circulation, undergoes minimal metabolism, and is largely excreted unchanged into the bile, leading to faecal elimination. Research has demonstrated that an *ABCG2* genetic variant increases the plasma concentration of simvastatin^121^ and is linked to a greater risk of SIM^122^. According to previous studies, SNPs in *ABCG2* decrease ABCG2 expression^123,124^.

CYP2C9 is responsible for the metabolism of certain statins, such as fluvastatin, pitavastatin, and rosuvastatin^125^. Genetic variations in *CYP2C9* affect the ability of an individual to metabolise these drugs. The two most extensively studied variants, CYP2C9*2 and CYP2C9*3, significantly metabolise statins more slowly, leading to higher drug levels and an increased risk of side effects. *CYP2C9* genetic variations have been shown to impact statin exposure and the risk of SAMS^109^.

Serum lipid levels and the response to statin therapy vary significantly among individuals^126^. While the data suggest that HMG-CoA reductase genotypes explain less than 2% of the variance in statin response, 6–15% of the variants in the gene encoding HMG-CoA reductase can be explained by alternative splicing^127,128^. An SNP located in intron 13 of the HMG-CoA reductase gene causes an alternate splicing event that involves exon 13 within the catalytic domain of the enzyme to inhibit its activity^118^. Alternative splicing polymorphisms of HMG-CoA reductase have a significant effect on the response of women to statin therapy, according to a study of a group of patients with familial hypercholesterolaemia^129^.

The type and quantity of *LDLR* mutations affect the patient’s response to lipid-lowering medications. Numerous randomised trials have shown that the risk of cardiovascular events is decreased by treatments that lower LDL-C levels by reducing LDL particles through upregulation of LDLR^130,131^. Recent data suggest that berberine-mediated LDL-C reduction depends on the 3′-untranslated region (3-UTR) of LDLR, which stabilises LDLR mRNA. SNPs in the 3-UTR are linked to both in vitro LDLR mRNA stability and in vivo LDL-C levels^132^. Additionally, Polisecki et al. reported that variations in this region might affect statin-mediated lipid reduction. Certain variations in *LDLR* have been linked to lipid-lowering responses following simvastatin administration^133^.

The main metabolic pathway of statins, including atorvastatin and simvastatin, is the CYP3A4 pathway^134^. Inducers or inhibitors of this enzyme can change the plasma concentration of statins, which may reduce the effectiveness of treatment or increase the risk of side effects such as myopathy^135^. It has been demonstrated that groups of affected individuals can differ in CYP3A4 activity by up to ten times, possibly due to differences in the genes encoding these enzymes. A study of patients with hypercholesterolaemia treated with atorvastatin revealed that the *CYP3A4* promoter variant rs2740574 is associated with elevated LDL-C levels. Compared to carriers of the wild-type allele, carriers of the missense variant rs4986910 showed increased efficacy and decreased LDL-C levels^136^. Another study showed no correlation between the rs4986910 variant and atorvastatin response^137^. The rs2740574 variant has been reported to significantly reduce LDL-C levels in individuals with wild-type genotypes compared to those with the variant allele.

The CPIC guidelines offer recommendations for the use of pharmacogenetic information, including statins, in clinical practice. Dose adjustment based on pharmacogenomics involves altering the dosage of a medication according to an individual’s genetic profile to enhance the effectiveness of the drug and reduce the risk of adverse effects^109^. CPIC guidelines state how *SLCO1B1, ABCG2, and CYP2C9* genotypes can guide statin therapy to reduce the risk of SAMS. Based on weak evidence and a lack of conclusive clinical applications, no recommendations have been provided for *CYP3A4/5* and *HMGCR*^109^.

## The pharmacogenetics of antihypertensive drugs

### The pharmacogenetics of β-blockers

CVDs such as ischaemic heart disease, hypertension, cardiac arrhythmias, and heart failure are frequently treated with β-adrenergic receptor blockers or β-blockers^138^. The activation of the adrenergic nervous system influences the onset and progression of numerous cardiovascular and associated metabolic disorders^139^. β-blockers produce negative inotropic (reduced force of myocardial contraction), negative dromotropic (reduced conduction velocity through the atrioventricular node), and chronotropic (reduced heart rate [HR]) effects by modulating activation of the sympathetic nervous system^138^. Numerous investigations have verified a strong correlation between the HR-lowering effect of β-blockers and advantageous cardiovascular consequences^140,141^.

#### The therapeutic target of β-blockers

β-adrenergic receptors play a significant role in cardiac function via signal transduction controlled by G protein-coupled receptor kinase (GPCRK) phosphorylation interactions. The therapeutic goal of β-blockers is to prevent the binding of a ligand (catecholamines, either norepinephrine or epinephrine) to β-adrenergic receptors (β1AR and β2AR) by competing for the binding site. After the agonist binds to the βAR, it is linked to the Gs protein (Gαs) to activate adenyl cyclase (AC) and produce cyclic AMP (cAMP), which further phosphorylates GRK2 and then activates PKA, which in turn phosphorylates various intracellular substrates for practical function^142^. The heart is the primary target of β-adrenergic receptor stimulation. When this receptor is activated, the contractility (inotropy), HR, and conduction velocity (homotopy) of the heart increase. The cardiovascular system can be better regulated by taking advantage of β1AR and β2AR signalling. When catecholamines overstimulate the β-adrenergic receptors, they cause CVD, stroke, heart failure, and cardiac enlargement. Elevating the level of Gα causes βAR kinase (βARK) to become active, which in turn influences and accelerates the development of heart failure by stimulating the cardiomyocyte βARs. Furthermore, in a practical sense, an increase in GRKs limits heart failure pathogenesis via the desensitisation of βARs to control overstimulation^142^.

#### Genetic polymorphisms affecting β-blockers response

Although β-blockers are generally effective, their HR-lowering effects vary among individuals^143^. Genetic factors have been identified as the cause of this variability in the HR^144^. Approximately 25% of medications undergo biotransformation, significantly aided by cytochrome P450 2D6 (CYP2D6). Several β-blockers, including metoprolol, carvedilol, propranolol, labetalol, nebivolol, and timolol, are substrates of the CYP2D6 enzyme^145^. Metoprolol is a primary β-blocker whose pharmacokinetic variability, largely due to its dependence on CYP2D6 for metabolism, may lead to significant differences in pharmacodynamic responses among individuals^146^. Many studies have shown that CYP2D6 poor and intermediate metabolizers exhibit decreased apparent oral clearance of metoprolol. These pharmacokinetic differences primarily result in variations in heart rate (HR) response; however, other CYP2D6 phenotypes do not appear to significantly affect the response to β-blockers^147–149^. The *CYP2D6* genotype is linked to a changed HR response to β-blocker therapy, and it has been demonstrated that the CYP2D6 phenotype is one of the most important indicators of variation in HR response to metoprolol^150^. Notably, the HR indicates the level of β1AR blockade when β-blocker therapy is administered. The correlations between HR and *CYP2D6* genotype were consistent, but the correlations between blood pressure and other responses were weaker^147^. However, some studies have shown a positive correlation between the *CYP2D6* genotype and blood pressure response^147,151,152^.

Sympathetic nervous system activation mediates chronotropic, dromotropic, and inotropic effects through β1AR, which are GPCRs encoded by adrenoceptor beta 1 (*ADRB1*). These receptors are mainly expressed in cardiac tissue^153^. The *ADRB1* variants rs1801252 (*ADRB1* 145 A > G; Ser49Gly) and rs1801253 (*ADRB1* 1165 C > G; Arg389Gly) are the most prevalent and well-studied. The variant rs1801252 causes agonist-promoted downregulation, whereas the variant rs1801253 modifies G-protein coupling and G-protein-coupled cyclase activity and decreases cAMP production^154,155^. The strongest evidence points to a relationship between the *ADRB1* genotype and reduced diastolic blood pressure observed in response to β-blockers. However, the *ADRB1* genotype was not associated with changes in HR or systolic blood pressure. Additionally, the data indicated that patients with the *ADRB1* haplotype would likely benefit the most from β-blocker therapy and have a lower chance of developing MACEs and chronic heart failure^147^.

β2AR is a G protein-coupled adrenergic receptor that is encoded by *ADRB2*. Vascular smooth muscle cells, bronchial smooth muscle cells, and cardiac myocytes express β2AR. The *ADRB2* (46 A > G; Arg16Gly) SNP rs1042713 and *ADRB2* (79 C > G; Gln27Glu) SNP rs1042714 are two common SNPs. The rs1042713 variant amplified the downregulation induced by agonists, which in turn led to a reduction in cAMP formation. Conversely, the variant rs1042714 exhibits resistance against agonist-induced downregulation, which ultimately leads to an increase in the formation of cAMP^156,157^. Recent data from individuals with CAD and chronic heart failure collectively indicated that the *ADRB2* genotype is not associated with cardiovascular events^147,158^.

GPCRKs desensitise GPCRs that are occupied by ligands such as βARs, which eventually results in the downregulation of the receptor^153^. G protein-coupled receptor kinase 5 (GPCRK5) is highly expressed in cardiac tissue. The *GPCRK5* Leu41 variant encodes a functional kinase with increased in vitro desensitisation of β1AR, which reduces receptor stimulation and cAMP generation^159,160^. For *GPCRK5* variants, the homozygous genotype with the major allele (Gln41Gln) was linked to a better response to β-blockers during treatment. In contrast, carriers of the Leu41 allele do not benefit from β-blocker therapy^147^.

*ADRA2C* encodes α2C AR, a presynaptic GPCR that reduces the release of norepinephrine from sympathetic nerves upon activation. α2ARs inhibit adenylyl cyclase in response to endogenous catecholamine stimulation, which lowers cAMP levels and causes noradrenergic neurons to become hyperpolarized^161^. Individuals of African heritage are most likely to have the in-frame deletion rs61767072 (c.971_982del), which results in the loss of four amino acids (p.Gly324_Ala327del, sometimes called Del322-325), and is associated with decreased receptor function^162^. This leads to increased sympathetic nervous activity and an increased catecholamine response to the α2AR antagonist, yohimbine^163^.

Fewer studies are available related to individual variants of *ADRA2C, GPCRK4*, and *GPCRK5* and their associations with β-blocker responses. Therefore, there is insufficient evidence to provide therapeutic recommendations for the individual variants of *CYP2D6, ADRB2, ADRA2C, GPCRK4* and *GPCRK5*^164^.

### The pharmacogenetics of angiotensin-converting enzyme inhibitors

Angiotensin-converting enzyme inhibitors (ACEIs) are frequently used to treat common conditions, such as heart failure, chronic renal disease, and hypertension.

#### The therapeutic targets of ACE inhibitors

The therapeutic effect of ACE inhibitors is to reduce the amount of angiotensin II, which increases sodium excretion in the urine, lowers blood pressure, and prevents the remodelling of smooth muscle and cardiac muscle cells^165,166^. The renin-angiotensin-aldosterone system (RAAS) interacts with ACEIs. The RAAS controls blood pressure by releasing angiotensinogen from the liver into the circulation, where renin converts angiotensinogen to angiotensin I. Angiotensin I (Ang I) is converted to angiotensin II (Ang II) by ACE. Ang II attachment to angiotensin receptors promotes blood vessel constriction, which increases blood pressure. Furthermore, Ang II stimulates aldosterone synthesis, which increases the ability of the blood to reabsorb salt and water and elevates blood pressure. ACEIs limit smooth muscle constriction in the vasculature and decrease aldosterone levels by blocking the ability of ACE to convert Ang I to Ang II and by lowering blood pressure. ACE is an essential enzyme involved in bradykinin degradation. Bradykinin builds up under conditions of ACE inhibition, and increased bradykinin levels may have side effects such as cough and angioedema^167^.

#### Genetic polymorphisms affecting ACE inhibitor response

Genes involved in the renin-angiotensin pathway are among those extensively studied in the pharmacogenetics of ACEIs. One of the most widely investigated genetic variants is the insertion/deletion (I/D) polymorphism in the ACE gene, characterised by the presence (insertion, I) or absence (deletion, D) of a 287-base pair Alu repeat sequence within intron 16. This polymorphism results in three genotypes: II, ID, and DD. The D allele is associated with elevated ACE enzyme activity and higher circulating ACE levels, whereas the I allele corresponds to lower ACE activity. These enzymatic differences can affect individual responses to ACEIs, which are commonly prescribed for hypertension and heart failure^168–170^. The distribution of the I and D alleles varies significantly across populations. In European populations, the alleles are relatively evenly distributed. In contrast, African populations tend to exhibit a higher frequency of the D allele, for example, ~70% in Morocco, with similar trends observed across the continent. Middle Eastern populations, including Saudi Arabia, also show a high prevalence of the D allele, estimated at around 72.5%. Conversely, East Asian populations such as those in China and Japan demonstrate a greater prevalence of the I allele, with frequencies ranging from 65 to 70%. These population-based differences underscore the potential role of genetic background in influencing both the efficacy and safety of ACEI therapy, reinforcing the importance of incorporating genetic factors into personalised treatment strategies^171^.

Genetic variations at this locus correlate with hypertension^172,173^ and myocardial infarction^174,175^. As the presence of the *ACE* I/D gene polymorphisms explained more than half of the total phenotypic variance in ACE activity, it was demonstrated to have a strong correlation with plasma ACE levels. Studies have shown that individuals with genotype II have lower ACE concentrations than those with DD^176^. The DD genotype has been associated with increased ACE activity and levels, which increase the production of Ang II and ultimately elevate blood pressure^177^. In a study of male Malaysian hypertensive participants, those with the DD genotype showed a more robust response to ACEIs (lisinopril and enalapril)^178^.

Angiotensinogen (AGT), a primary structural gene in the RAAS pathway, is associated with hypertension^179^. Genetic variations in *AGT* lead to alterations in plasma AGT concentrations, which may contribute to the development of hypertension, CAD, and myocardial infarction^180^. The *AGT* SNPs M235T (rs699), M174T (rs4762) and A-6G (rs5051) are associated with serum AGT levels and hypotension in response to ACEIs^181,182^. Other studies have not found this association^183^. However, there was a strong correlation between hypertension and the T allele of M235T, which was linked to higher plasma AGT levels^184^. *AGT* SNPs (T174M and M235T) are among the most prevalent variants that cause hypertension in several populations^180^. There are other variants, such as G-217A, A-6G, A-20C and G-152A; however, their significance is not as great as that of variants T174M and M235T^180^.

The primary angiotensin II receptor AGTR1 mediates most of the physiological effects of AGT II, such as aldosterone secretion, pressure responses, renal tubular salt transport, and vascular contraction^185^. There is conflicting evidence in earlier research linking the (A1166C) *AGTR1* polymorphism to cardiovascular disorders. According to a previous study, the AA genotype protects against CAD and myocardial infarction, whereas the (1166C) *AGTR1* allele is a genetic marker that predisposes individuals to these conditions^186^. Other studies have not supported these conclusions^187,188^. According to a recent study published in 2022, AC heterozygosity may lower the risk of heart failure associated with *AGTR1* polymorphism^189^. Previous research findings have indicated that there is no correlation between *AGTR1* polymorphisms and response to ACEIs^190,191^. Frazier found that *AGTR1* polymorphisms are linked to blood pressure response to antihypertensive drugs in black women^192^. Moreover, another study revealed that hypertensive Chinese individuals with *AGTR1* polymorphism had lower blood pressure when treated with ACEIs^193^.

Common adverse effects of ACEIs, such as enalapril, have been associated with specific SNPs that influence their incidence. Enalapril, a prodrug targeting the RAAS, is rapidly converted to its active metabolite, enalaprilat. Among its side effects, a persistent dry cough is the most frequently reported. Genetic variations in the *SLCO1B1* gene, which encodes the hepatic transporter OATP1B1, have been linked to reduced hepatic clearance of enalapril, resulting in elevated plasma concentrations. This accumulation increases bradykinin levels, thereby contributing to the incidence of cough. Furthermore, polymorphisms in the *BDKRB2* gene, which encodes the bradykinin B2 receptor, have also been implicated in heightened susceptibility to ACEI-induced cough^194^.

According to a genome-wide association study (GWAS), mutations in potassium voltage-gated channel interacting protein 4 (*KCNIP4*) have also been linked to ACEI-induced cough^195^. The control of Kv4 potassium channels is one of the main functions of KCNIP4. Bradykinin buildup in the lungs can stimulate sensory nerve afferents when there is a mutation in the *KCNIP4* gene, which causes problems in Kv4 channel regulation^196^. Therefore, patients with *KCNIP4* polymorphisms that affect the regulation of Kv4 channels are more susceptible to the negative effects of ACEIs, because the resulting mutant proteins can stop bradykinin breakdown.

Angioedema is another potentially fatal side effect of ACEIs. Variants in immune-regulating genes (*ETV6*) and protein kinase C (*PRKCQ*) are risk factors for the side effects of ACEI, including angioedema. According to GWAS, *PRKCQ* variation is associated with a lower incidence of angioedema. In contrast, variations in *ETV6* are linked to a greater risk of angioedema associated with ACEIs^197^. Angioedema is strongly linked to variations in the membrane metalloendopeptidase (*MME*), which encodes neprilysin. Neprilysin is a secondary enzyme that degrades vasoactive peptides such as bradykinin in conjunction with ACE. Mutations in *MME* may lead to decreased neprilysin function, which causes an increase in vasoactive peptides in the plasma under ACE-inhibition conditions. Vasoactive peptide build-up can lead to a greater frequency of coughing and angioedema. Thus, ACEI adverse events are more likely to occur in patients with reduced neprilysin levels^197^.

## Discussion

Pharmacogenetics has become crucial for optimising CVD treatment, potentially reducing adverse drug reactions, and enhancing therapeutic efficacy. This review highlights significant advancements in our understanding of how genetic polymorphisms can influence CVD drug metabolism, transport, and therapeutic targets. The various prime-time genetic variants influencing cardiovascular drug responses discussed here include *CYP2C19* polymorphisms affecting clopidogrel drug response, *CYP2C9* and *VKORC1* polymorphisms impacting warfarin dose, *SLCO1B1* variants associated with statin-induced myopathy risk, and *CYP2D6* polymorphisms influencing β-blocker metabolism, particularly for metoprolol. Additionally, ACE insertion/deletion (I/D) polymorphisms affect responsiveness to ACE inhibitors. Emerging or preliminary variants, on the other hand, refer to those recently identified or still under investigation, such as the *MALL* gene linked to warfarin response, *EYA1* associated with aspirin-induced ulceration, and *KCNIP4* linked to ACE inhibitor-induced cough. While these variants show promise, further research is needed to establish their clinical relevance. Healthcare professionals should recognise that while ‘prime-time’ variants can currently guide clinical decisions, the identification of emerging variants highlights the evolving nature of pharmacogenetics, which may hold significant clinical implications in the future.

Pharmacogenetic testing for drugs such as clopidogrel, warfarin, statins, and β-blockers enables more precise genotype-guided treatments. However, significant gaps remain in the integration of pharmacogenetic knowledge into routine clinical practice. For example, while *CYP2C19* testing is well-documented in relation to clopidogrel response, its clinical adoption varies due to factors such as cost, limited access to genetic testing, and ongoing debates about its relevance across diverse populations. Additionally, research on non-European populations remains underrepresented, raising concerns about the broader applicability of pharmacogenetic findings across different ethnic groups. These disparities underscore the need for more inclusive studies to develop population-specific recommendations.

Innovative areas such as polygenic risk scores, machine-learning-based pharmacogenomic models, and next-generation sequencing offer promising avenues for identifying new biomarkers and treatment targets. To better understand genetic variability in drug responses, future research must integrate pharmacogenetic data with other omics approaches, including proteomics and metabolomics. The widespread implementation of personalised medicine in cardiovascular care depends on overcoming logistical and regulatory barriers, such as the affordability of genetic testing and the education of healthcare providers. While pharmacogenetics has significantly advanced our understanding of the variability in drug responses, fully incorporating it into clinical practice necessitates further investigation, infrastructure development, and collaboration among key stakeholders. Addressing these challenges will allow us to maximise the potential of pharmacogenetics in improving cardiovascular outcomes worldwide.
